# Factors Associated with Severe Outcomes Among Immunocompromised Adults Hospitalized for COVID-19 — COVID-NET, 10 States, March 2020–February 2022

**DOI:** 10.15585/mmwr.mm7127a3

**Published:** 2022-07-08

**Authors:** Jason Robert C. Singson, Pam Daily Kirley, Huong Pham, Gretchen Rothrock, Isaac Armistead, James Meek, Evan J. Anderson, Libby Reeg, Ruth Lynfield, Susan Ropp, Alison Muse, Christina B. Felsen, Melissa Sutton, H. Keipp Talbot, Fiona P. Havers, Christopher A. Taylor, COVID-NET Surveillance Team, Arthur Reingold, Shua J. Chai, COVID-NET Surveillance Team, Nisha B. Alden, Kim Yousey-Hindes, Kyle P. Openo, Erica Bye, Mark A. Montoya, Grant Barney, Kevin Popham, Nasreen Abdullah, William Schaffner

**Affiliations:** ^1^California Emerging Infections Program, Oakland California; ^2^Council of State and Territorial Epidemiologists, Atlanta, Georgia; ^3^CDC COVID-19 Emergency Response Team; ^4^Colorado Department of Public Health and Environment; ^5^Connecticut Emerging Infections Program, Yale School of Public Health, New Haven, Connecticut; ^6^Georgia Emerging Infections Program, Georgia Department of Health; ^7^Emory University School of Medicine, Atlanta, Georgia; ^8^Atlanta Veterans Affairs Medical Center, Atlanta, Georgia; ^9^Michigan Department of Health and Human Services; ^10^Minnesota Department of Health; ^11^New Mexico Department of Health; ^12^New York State Department of Health; ^13^University of Rochester School of Medicine and Dentistry, Rochester, New York; ^14^Public Health Division, Oregon Health Authority; ^15^Vanderbilt University Medical Center, Nashville, Tennessee; ^16^Career Epidemiology Field Officer Program, CDC.; Colorado Department of Public Health and Environment; Connecticut Emerging Infections Program, Yale School of Public Health, New Haven, Connecticut; Emory University School of Medicine, Atlanta, Georgia; Georgia Emerging Infections Program, Georgia Department of Health; Atlanta Veterans Affairs Medical Center, Atlanta, Georgia; Minnesota Department of Health; New Mexico Department of Health; New York State Department of Health; University of Rochester School of Medicine and Dentistry, Rochester, New York; Public Health Division, Oregon Health Authority; Vanderbilt University Medical Center, Nashville, Tennessee.

Immunocompromised persons are at increased risk for severe COVID-19–related outcomes, including intensive care unit (ICU) admission and death (*1*). Data on adults aged ≥18 years hospitalized with laboratory-confirmed COVID-19 from 10 U.S. states in the COVID-19–Associated Hospitalization Surveillance Network (COVID-NET) were analyzed to assess associations between immunocompromise and ICU admission and in-hospital death during March 1, 2020–February 28, 2022. Associations of COVID-19 vaccination status with ICU admission and in-hospital death were also examined during March 1, 2021–February 28, 2022. During March 1, 2020–February 28, 2022, among a sample of 22,345 adults hospitalized for COVID-19, 12.2% were immunocompromised. Among unvaccinated patients, those with immunocompromise had higher odds of ICU admission (adjusted odds ratio [aOR] = 1.26; 95% CI = 1.08–1.49) and in-hospital death (aOR = 1.34; 95% CI = 1.05–1.70) than did nonimmunocompromised patients. Among vaccinated patients,* those with immunocompromise had higher odds of ICU admission (aOR = 1.40; 95% CI = 1.01–1.92) and in-hospital death (aOR = 1.87; 95% CI = 1.28–2.75) than did nonimmunocompromised patients. During March 1, 2021–February 28, 2022, among nonimmunocompromised patients, patients who were vaccinated had lower odds of death (aOR = 0.58; 95% CI = 0.39–0.86) than did unvaccinated patients; among immunocompromised patients, odds of death between vaccinated and unvaccinated patients did not differ. Immunocompromised persons need additional protection from COVID-19 and using multiple known COVID-19 prevention strategies,^†^ including nonpharmaceutical interventions, up-to-date vaccination of immunocompromised persons and their close contacts,^§^ early testing, and COVID-19 prophylactic (Evusheld) and early antiviral treatment,^¶^ can help prevent hospitalization and subsequent severe COVID-19 outcomes among immunocompromised persons.

COVID-NET is a CDC-funded collaboration for population-based surveillance of laboratory-confirmed COVID-19–associated hospitalization in 99 U.S. counties in 14 states. A COVID-NET case is defined as a positive real-time reverse transcription–polymerase chain reaction or rapid antigen test result for SARS-CoV-2 (the virus that causes COVID-19) within 14 days before or during hospitalization in a person who lived in the surveillance catchment area. Medical chart abstraction and representative sampling methods have been described previously (*2*). Data collected on sampled adults hospitalized during March 1, 2020–February 28, 2022, across 10 participating states** were examined. Patients whose hospitalization was not likely related to COVID-19^††^ and those without a completed chart review were excluded. Immunocompromised patients were defined as those having one or more predefined immunocompromising conditions.^§§^ COVID-19 vaccination definitions for immunocompromised persons changed during the study period^¶¶^; in this analysis, a vaccinated patient was defined as one who had received both doses of a 2-dose COVID-19 vaccination series or 1 dose of a single-dose COVID-19 vaccine with or without additional or booster doses ≥14 days before their positive SARS-CoV-2 test result, per state immunization information system records. Vaccinated patients with additional or booster doses were not analyzed separately. Patients were considered unvaccinated if no COVID-19 vaccination was recorded before the positive test result; patients who were documented to have received only the first dose of a 2-dose series or their last vaccination series dose <14 days before receiving a positive SARS-CoV-2 test result were excluded.

Demographic and clinical characteristics of hospitalized patients were assessed; Pearson’s chi-square tests were used to compare differences between immunocompromised and nonimmunocompromised patients. Bivariate and multivariable logistic regression analyses were used to assess associations between immunocompromise and both ICU admission and in-hospital death among vaccinated and unvaccinated patients in separate models. Associations between each individual immunocompromising condition and death were assessed using multivariable analyses, adjusting only for age and sex to improve model convergence. Bivariate and multivariable analyses were used to assess the association between vaccination status and both ICU admission and in-hospital death among immunocompromised and nonimmunocompromised patients in separate models, using data beginning March 1, 2021, when immunocompromised patients first reported receiving vaccine doses, through February 28, 2022. Multivariable analyses were adjusted for age, sex, site (entered as a fixed effect), SARS-CoV-2 variant–predominant period,*** and other factors with documented or potential association and a p-value <0.10 in bivariate analyses. Statistical analyses used SAS (version 9.4; SAS Institute) survey procedures to account for sampling weights, with statistical significance set at alpha = 0.05. This activity was reviewed by CDC and conducted consistent with applicable federal law and CDC policy.^†††^

During March 1, 2020–February 28, 2022, a representative sample of 24,625 (11.0%, unweighted) of 223,069 COVID-NET cases had complete chart review, including 22,345^§§§^ (90.7%, unweighted) that met inclusion criteria. Among the 22,345 patients included, 12.2% were immunocompromised, including 11.1%, 10.9%, and 17.3% of patients hospitalized during the pre-Delta, Delta, and Omicron variant–predominant periods, respectively. Overall, immunocompromised patients were more likely to be older and to be non-Hispanic White (Table 1). Compared with nonimmunocompromised patients, those with immunocompromise had a statistically significantly higher prevalence of all underlying medical conditions except diabetes and neurologic disease.

**TABLE 1:** Demographic and clinical characteristics of adults hospitalized for laboratory-confirmed COVID-19 (N = 22,345), by immunocompromise status and vaccination status* — COVID-NET, 10 states,^†^ March 1, 2020–February 28, 2022

Characteristic	Immunocompromised, no. (weighted %)^§^								
	Overall			Unvaccinated			Vaccinated^†^		
	Yes	No	p-value	Yes	No	p-value	Yes	No	p-value
**Total**	**2,209 (100.0)**	**20,136 (100.0)**	**—**	**1,855 (100.0)**	**18,825 (100.0)**	**—**	**354 (100.0)**	**1,311 (100.0)**	**—**
**Age group, yrs**									
18–49	492 (17.3)	6,509 (24.9)	<0.01	447 (18.1)	6,336 (26.9)	<0.01	45 (15.5)	173 (12.1)	0.09
50–64	717 (28.2)	6,298 (29.4)		625 (31.7)	5,932 (30.3)		92 (20.1)	366 (23.7)	
65–74	472 (25.8)	3,192 (19.5)		377 (24.5)	2,915 (18.9)		95 (28.6)	277 (23.6)	
75–84	378 (20.8)	2,501 (15.6)		296 (18.5)	2,213 (14.1)		82 (26.3)	288 (25.2)	
≥85	150 (7.9)	1,636 (10.6)		110 (7.2)	1,429 (9.8)		40 (9.5)	207 (15.5)	
**Race or ethnicity^¶^**									
White	1,219 (52.4)	9,587 (47.7)	<0.01	987 (52.7)	8,711 (45.7)	<0.01	232 (51.7)	876 (60.0)	0.46
Black	496 (25.5)	4,340 (23.2)		440 (26.5)	4,142 (24.1)		56 (23.1)	198 (18.2)	
AI/AN	34 (1.1)**	452 (2.0)**		30 (1.2)**	431 (2.0)**		4 (1.1)**	21 (1.7)**	
A/PI	91 (4.8)	1,201 (5.1)		81 (4.3)**	1159 (5.4)		10 (6.1)**	42 (3.6)**	
Hispanic	301 (12.7)	3,803 (16.5)		264 (13.0)	3,678 (17.6)		37 (12.0)	125 (10.1)	
Other/Unknown^††^	68 (3.4)	753 (5.5)		53 (2.3)	704 (5.4)		15 (6.1)**	49 (6.4)	
**Sex**									
Male	1,121 (54.3)	10,819 (52.3)	0.25	931 (52.4)	10,145 (52.4)	0.98	190 (58.7)	674 (51.6)	0.17
Female	1,088 (45.7)	9,317 (47.7)		924 (47.5)	8,680 (47.6)		164 (41.3)	637 (48.4)	
**Resident of long-term care facility**	172 (8.7)	1,652 (10.1)	0.16	150 (9.4)	1448 (9.4)	0.99	22 (7.1)	204 (14.6)	0.06
**Variant predominance** ^§§^									
Pre-Delta	1,730 (54.2)	16,654 (60.3)	<0.01	1,646 (74.2)	16,350 (69.1)	<0.01	84 (7.1)	304 (5.8)	0.20
Delta	318 (20.0)	2,683 (22.6)		154 (14.3)	2,047 (20.1)		164 (33.4)	636 (38.3)	
Omicron	161 (25.8)	799 (17.1)		55 (11.4)	428 (10.8)		106 (59.5)	371 (55.9)	
**Vaccination status^†^**									
Unvaccinated	1,855 (70.1)	18,825 (86.1)	<0.01	1,855 (100.0)	18,825 (100.0)	NA	NA	NA	NA
Vaccinated, without booster or additional doses	298 (21.3)	1,186 (11.6)		NA	NA		298 (71.4)	1,186 (83.3)	<0.01
Vaccinated, with booster or additional doses	56 (8.5)	125 (2.3)		NA	NA		56 (28.6)	125 (16.7)	
**Type of immunocompromising condition**									
AIDS or CD4+ count <200	37 (1.3)	NA	NA	33 (1.4)	NA	NA	4 (0.9)**	NA	NA
Complement deficiency	4 (0.1)**	NA	NA	4 (0.2)**	NA	NA	NA	NA	NA
Graft versus host disease	7 (0.3)**	NA	NA	7 (0.4)**	NA	NA	NA	NA	NA
HIV infection	177 (6.7)	NA	NA	159 (7.7)	NA	NA	18 (4.2)	NA	NA
Immunoglobulin deficiency/ Immunodeficiency	48 (1.8)	NA	NA	44 (1.6)	NA	NA	4 (2.4)**	NA	NA
Immunosuppressive therapy	664 (32.2)	NA	NA	529 (28.3)	NA	NA	135 (41.4)	NA	NA
Leukemia	135 (6.6)	NA	NA	111 (6.9)	NA	NA	24 (5.8)**	NA	NA
Lymphoma (Hodgkin or non-Hodgkin)	125 (5.9)	NA	NA	96 (5.5)	NA	NA	29 (6.9)	NA	NA
Metastatic cancer	212 (11.0)	NA	NA	172 (11.1)	NA	NA	40 (10.6)	NA	NA
Multiple myeloma	52 (2.7)	NA	NA	37 (2.1)	NA	NA	15 (3.9)**	NA	NA
Solid organ malignancy	791 (37.2)	NA	NA	649 (34.2)	NA	NA	142 (44.2)	NA	NA
Steroid therapy	610 (26.5)	NA	NA	533 (30.4)	NA	NA	77(17.2)	NA	NA
Transplant, hematopoietic stem cell	26 (1.3)**	NA	NA	20 (0.8)**	NA	NA	6 (2.7)**	NA	NA
Transplant, solid organ	253 (14.1)	NA	NA	195 (10.6)	NA	NA	58 (22.2)	NA	NA
**Underlying medical condition**									
Any underlying medical condition^†††^	2,097 (94.5)	17,888 (90.3)	<0.01	1,758 (94.2)	16,643 (89.5)	<0.01	339 (95.4)	1245 (95.1)	0.87
Hypertension	1,374 (67.0)	10,649 (58.2)	<0.01	1,125 (64.1)	9,732 (56.2)	<0.01	249 (74.0)	917 (70.6)	0.30
Diabetes mellitus	738 (37.5)	6,745 (35.2)	0.05	599 (35.5)	6,212 (34.4)	0.39	139 (42.0)	533 (40.2)	0.66
Chronic lung disease	868 (39.1)	5,674 (29.0)	<0.01	726 (40.2)	5,146 (27.1)	<0.01	142 (36.4)	528 (41.1)	0.15
Chronic metabolic (except diabetes)	380 (18.1)	2,469 (13.9)	<0.01	307 (16.9)	2,212 (13.3)	<0.01	73 (20.8)	257 (17.3)	0.11
Cardiovascular disease	1,043 (52.1)	6,629 (38.8)	<0.01	838 (52.8)	5,894 (35.8)	<0.01	205 (50.5)	735 (57.4)	0.13
Liver disease	253 (11.5)	1,082 (5.6)	<0.01	200 (10.2)	966 (5.1)	<0.01	53 (14.5)	116 (8.2)	<0.01
Renal disease	575 (30.6)	2,816 (16.3)	<0.01	458 (26.7)	2,462 (14.6)	<0.01	117 (39.8)	354 (27.1)	<0.01
Blood disorder	195 (10.1)	555 (3.0)	<0.01	151 (9.0)	489 (2.6)	<0.01	44 (12.5)	66 (5.3) **	0.04
Neurologic disease	475 (22.5)	3,917 (20.2)	0.14	381 (19.8)	3,514 (18.4)	0.31	94 (29.0)	403 (31.8)	0.53
Rheumatologic/Autoimmune condition	542 (27.1)	707 (4.7)	<0.01	432 (25.1)	617 (4.1)	<0.01	110 (31.9)	90 (8.0)	<0.01
Obesity	951 (38.4)	9,823 (45.3)	<0.01	811 (40.2)	9276 (46.4)	<0.01	140 (34.1)	547 (38.5)	0.19
**No. of underlying conditions**									
0	112 (5.5)	2,248 (9.7)	<0.01	97 (5.8)	2,182 (10.5)	<0.01	15 (4.6)**	66 (4.9)	0.17
1	252 (9.3)	3,630 (15.9)		227 (11.3)	3,500 (17.1)		25 (4.8)	130 (8.5)	
2	371 (15.8)	4,356 (21.2)		323 (15.6)	4,149 (21.9)		48 (16.4)	207 (17.0)	
≥3	1,474 (69.3)	9,902 (53.2)		1,208 (67.3)	8,994 (50.6)		266 (74.2)	908 (69.7)	

**Abbreviations:** A/PI = Asian or Pacific Islander; AI/AN = American Indian or Alaska Native; COVID-NET = COVID-19–Associated Hospitalization Surveillance Network; NA = not applicable.

* Vaccinated patients were defined as those with a positive SARS-CoV-2 test result from a specimen collected ≥14 days after either the second dose of a 2-dose vaccination series or after 1 dose of a single dose vaccine. When not otherwise specified, vaccinated patients include those who might have received additional or booster doses. Vaccinated patients without additional or booster doses include both those eligible and not yet eligible for an additional or booster dose. Vaccinated patients with additional booster doses received additional or booster doses on or after August 13, 2021, with a positive SARS-CoV-2 test result from a specimen collected ≥14 days after receipt of the additional or booster dose.

^†^ Selected counties in California, Colorado, Connecticut, Georgia, Michigan, Minnesota, New Mexico, New York, Oregon, and Tennessee.

^§^ Representative sample of all cases reported to COVID-NET, stratified by patient age and COVID-NET site. Percentages were weighted to account for the probability of selection for sampled cases.

^¶^ White, Black, AI/AN, and A/PI persons were non-Hispanic; Hispanic persons could be of any race.

** Relative SE >30. Estimates might be unstable; results should be interpreted with caution.

^††^ Includes patients who were classified as multiracial, non-Hispanic. Non-Hispanic ethnicity was assumed for patients with unknown ethnicity.

^§§^ Pre-Delta variant–predominant period = March 1, 2020–June 26, 2021; Delta variant–predominant period = June 27–December 18, 2021; Omicron variant–predominant period = December 19, 2021–February 28, 2022. https://www.cdc.gov/mmwr/volumes/71/wr/mm7116e1.htm

^†††^ Defined as one or more of the following: chronic lung disease (including asthma), chronic metabolic disease, diabetes mellitus, blood disorder/hemoglobinopathy, cardiovascular disease, neurologic disease, renal disease, gastrointestinal/liver disease, rheumatologic/autoimmune condition, obesity, feeding tube dependence, or wheelchair dependence.

Among unvaccinated patients, those who were immunocompromised had higher odds of ICU admission (aOR = 1.26) and death (aOR = 1.34) than did nonimmunocompromised patients^¶¶¶^ (Table 2). Similarly, among vaccinated patients, those who were immunocompromised also had higher odds of ICU admission (aOR = 1.40) and in-hospital death (aOR = 1.87) compared with nonimmunocompromised patients.**** Among patients with a specific immunocompromising condition compared with patients without that condition (irrespective of immunocompromise status), the odds of in-hospital death were higher for those with AIDS or low CD4+ count (aOR = 2.03), immunosuppressive therapy use (aOR = 1.65), multiple myeloma (aOR = 5.28), or solid organ transplant (aOR = 2.12) and lower for patients with immunoglobulin deficiency (aOR = 0.16) (Supplementary Table 1, https://stacks.cdc.gov/view/cdc/118606).

**TABLE 2:** Association of immunocompromise status with intensive care unit admission and in-hospital death among patients hospitalized for COVID-19, by vaccination status* — COVID-NET, 10 states,^†^ March 1, 2020–February 28, 2022

Immunocompromised	No. (weighted %)^§^											
	Unvaccinated^¶^						Vaccinated*^,^**					
	ICU admission			Death			ICU admission			Death		
	Yes	No	aOR (95% CI)	Yes	No	aOR (95% CI)	Yes	No	aOR (95% CI)	Yes	No	aOR (95% CI)
Yes	533 (26.6)	1,322 (73.4)	1.26 (1.08–1.49)^††^	272 (14.5)	1,582 (85.5)	1.34 (1.05–1.70)^§§^	85 (25.0)	269 (75.0)	1.40 (1.01–1.92)^§§^	55 (16.5)	298 (83.5)	1.87 (1.28–2.75)^††^
No	4,884 (22.8)	13,875 (77.2)	Ref	1,881 (11.0)	16,906 (89.0)	Ref	257 (18.7)	1,047 (81.3)	Ref	114 (9.6)	1,190 (90.4)	Ref

**Abbreviations:** aOR = adjusted odds ratio; COVID-NET = COVID-19–Associated Hospitalization Surveillance Network; ICU = intensive care unit; Ref = referent group.

* Vaccinated patients were defined as those with a positive SARS-CoV-2 test result from a specimen collected ≥14 days after the second dose of a 2-dose vaccination series or after 1 dose of a single dose vaccine. When not otherwise specified, vaccinated patients include those who might have received additional or booster doses.

^†^ Selected counties in California, Colorado, Connecticut, Georgia, Michigan, Minnesota, New Mexico, New York, Oregon, and Tennessee.

^§^ Representative sample of all cases reported to COVID-NET, stratified by age and COVID-NET site. Percentages were weighted to account for the probability of selection for sampled cases.

^¶^ ICU admission model among unvaccinated patients was adjusted for age, sex, site, variant predominant period, race/ethnicity, hypertension, diabetes, chronic lung disease, cardiovascular disease, renal disease, and obesity. ICU status was not known for 66 nonimmunocompromised patients. Death model among unvaccinated patients was adjusted for age, sex, site, variant predominant period, race/ethnicity, long-term care facility, hypertension, diabetes, chronic lung disease, cardiovascular disease, renal disease, blood disorders, neurologic disease, and rheumatologic/autoimmune condition. Death outcome was unknown for one immunocompromised patient and 38 nonimmunocompromised patients.

** ICU admission model among vaccinated patients was adjusted for age, sex, site, variant predominant period, race/ethnicity (American Indian or Alaska Native and Asian or Pacific Islander were reclassified as other/unknown because of small numbers as well as patients who identified as multiracial or unknown race), chronic metabolic disease, liver disease, and rheumatologic/autoimmune condition. ICU status was not known for seven nonimmunocompromised patients. Death model among vaccinated patients was adjusted for age, sex, site, variant predominant period, race/ethnicity, cardiovascular disease, renal disease, and rheumatologic/autoimmune condition. Death outcome was unknown for one immunocompromised patient and seven nonimmunocompromised patients.

^††^ p-value <0.01.

^§§^ p-value <0.05.

Among immunocompromised patients, those who were vaccinated did not have statistically significantly different odds of ICU admission or in-hospital death^††††^ compared with unvaccinated patients (Table 3). Among nonimmunocompromised patients, those who were vaccinated had lower odds of death (aOR = 0.58) than did unvaccinated patients.^§§§§^

**TABLE 3:** Association of vaccination status* with intensive care unit admission and in-hospital death among patients hospitalized for COVID-19, by immunocompromise status—COVID-NET, 10 states,^†^ March 1, 2021–February 28, 2022

Vaccination status*	No. (weighted %)^§^											
	Immunocompromised^¶^						Not immunocompromised**					
	ICU admission			Death			ICU admission			Death		
	Yes	No	aOR (95% CI)	Yes	No	aOR (95% CI)	Yes	No	aOR (95% CI)	Yes	No	aOR (95% CI)
**Vaccinated**	85 (25.0)	269 (75.0)	1.01 (0.64–1.58)	55 (16.5)	298 (83.5)	1.34 (0.71–2.51)	257 (18.7)	1,044 (81.3)	0.85 (0.60–1.12)	113 (9.5)	1,188 (90.5)	0.58 (0.39–0.86)^††^
**Unvaccinated**	129 (25.5)	351 (74.5)	Ref	66 (12.9)	413 (87.1)	Ref	1,121 (21.6)	3,771 (78.4)	Ref	488 (10.1)	4,409 (89.9)	Ref

**Abbreviations:** aOR = adjusted odds ratio; COVID-NET = COVID-19–Associated Hospitalization Surveillance Network; ICU = intensive care unit; Ref = referent group.

* Vaccinated patients were defined as those with a positive SARS-CoV-2 test result from a specimen collected ≥14 days after either the second dose of a 2-dose vaccination series or after 1 dose of a single dose vaccine. When not otherwise specified, vaccinated patients include those who might have received additional or booster doses.

^†^ Selected counties in California, Colorado, Connecticut, Georgia, Michigan, Minnesota, New Mexico, New York, Oregon, and Tennessee.

^§^ Representative sample of all cases reported to COVID-NET, stratified by age and COVID-NET site. Percentages were weighted to account for the probability of selection for sampled cases.

^¶^ ICU admission model among immunocompromise patients was adjusted for age, sex, site, variant predominant period, rheumatologic/autoimmune condition, and obesity. Death model among immunocompromised patients was adjusted for age, sex, site, variant predominant period hypertension, renal disease, and rheumatologic/autoimmune condition. Death outcome among immunocompromised patients was not known for one unvaccinated patient and one vaccinated patient without additional or booster doses.

** ICU admission model among nonimmunocompromised patients was adjusted for age, sex, site, variant predominant period, diabetes, and obesity. Death model among nonimmunocompromised patients was adjusted for age, sex, site, variant predominant period, long-term care facility residence, hypertension, diabetes mellitus, chronic metabolic disease, cardiovascular disease, renal disease, and neurologic disease. Death outcome among nonimmunocompromised patients was not known for 11 unvaccinated patients, three patients vaccinated without additional or booster doses, and three patients vaccinated with additional or booster doses.

^††^ p-value <0.05.

During the pre-Delta and Delta variant–predominant periods, immunocompromised patients generally had higher odds of death, irrespective of vaccination status compared with nonimmunocompromised patients, and nonimmunocompromised patients who were vaccinated had lower odds of death compared with unvaccinated patients (Supplementary Table 2, https://stacks.cdc.gov/view/cdc/118607). However, in the Omicron variant–predominant period, odds of death, irrespective of immunocompromise or vaccination status, were not statistically significantly different.

## Discussion

Once hospitalized, immunocompromised patients with COVID-19 had increased odds of ICU admission or in-hospital death, irrespective of vaccination status, compared with nonimmunocompromised patients, after adjusting for differences in demographic and clinical characteristics. The generally consistent association of individual immunocompromising conditions with increased odds of death suggests that immunocompromise itself was likely associated with severe outcomes.

COVID-19 vaccination among immunocompromised persons is highly protective against COVID-19–associated hospitalization (*3*), leading to fewer hospitalized patients who are then admitted to the ICU or die in-hospital. Once patients were hospitalized, however, vaccination status was not associated with ICU admission or death among immunocompromised patients in these analyses; patients with more medical conditions likely had closer medical follow-up and were strongly advised to be vaccinated, biasing vaccinated patients to be those at higher risk for severe outcomes, potentially contributing to the absence of observed differences. In addition, vaccine effectiveness against severe outcomes in immunocompromised persons is known to be lower than that in nonimmunocompromised persons (*3*,*4*). In comparison, nonimmunocompromised hospitalized patients who were vaccinated had reduced odds of death compared with those who were unvaccinated, consistent with the known protective effect of vaccination against severe outcomes in persons who can mount a robust immune response after vaccination. During the Omicron variant–predominant period, however, the effects of immunocompromise and vaccination on odds of death were attenuated in all patients, potentially due to the lower proportion of severe outcomes during hospitalization associated with this variant,^¶¶¶¶^ as well as the increased prevalence of previous infection-conferred immunity resulting in a decreased risk for infection across all groups and waning of vaccine-derived protection among those who received vaccine doses earlier in the COVID-19 pandemic. Because of these attenuated effects and the inability to further stratify by receipt of additional or booster doses because stratification generated unstable estimates (relative SE >30) in the analysis, the effect of additional or booster doses on death among immunocompromised patients was not able to be assessed.

Data from population-based, active surveillance suggest that immunocompromised adults are overrepresented among patients hospitalized with COVID-19 in the United States, accounting for 12.2% of adult hospitalizations in COVID-NET compared with an estimated 2.7% of the U.S. adult population (*5*). However, immunocompromised patients in COVID-NET shared similar demographic characteristics with the underlying U.S. noninstitutionalized immunocompromised population. The age ranges with the highest percentages of immunocompromised patients were similar (COVID-NET: 50–74 years; U.S. population: 50–69 years) (*5*). The older age distribution among immunocompromised patients likely contributed to their higher prevalences of underlying conditions known to be associated with poor COVID-19 outcomes, including chronic lung disease, renal disease, and obesity, which would increase the likelihood of severe COVID-19, hospitalization, and inclusion in this analysis (*6*).

The findings in this report are subject to at least four limitations. First, the analyses did not control for time since vaccination; earlier eligibility for and receipt of vaccines by immunocompromised patients might have resulted in earlier waning of protection, complicating identification of associations between vaccination and severe outcomes. Second, whereas the active, population-based nature of COVID-NET data minimizes the risk for capturing a nonrepresentative sample of hospitalized patients, clinicians might have admitted immunocompromised patients who were less ill than were nonimmunocompromised patients, leading to smaller observed differences in severe outcomes. Third, the number of immunocompromised persons within COVID-NET catchment areas is unknown; therefore, population-based rates of severe outcomes by immunocompromise and vaccination status not conditioned on hospitalization could not be calculated. Finally, changing recommendations and absence of data on prehospitalization prophylactic or treatment medications for COVID-19 limited the ability to account for treatments; severe outcomes might have been mitigated in patients who received these medications.

Given the increased odds of severe COVID-19 outcomes among immunocompromised hospitalized patients, multilayered prevention strategies for immunocompromised persons are critical to preventing hospitalization for COVID-19 and subsequent severe outcomes, especially when community levels indicate increased transmission and disease severity (*7*,*8*).***** These strategies include implementing nonpharmaceutical interventions; ensuring that immunocompromised persons and their close contacts are up to date with COVID-19 vaccination; urging immunocompromised persons to use effective preexposure prophylactic therapeutics, such as Evusheld; early testing, such as at-home tests; and early disease treatments, such as antiviral medications. Improved access to and use of these measures with considerations for socioeconomically disadvantaged and historically underserved racial and ethnic groups will help ensure health equity (*9*,*10*).^†††††^

**Summary**

**What is already known about this topic?**

Immunocompromise is associated with increased risk for intensive care unit (ICU) admission and in-hospital death after SARS-CoV-2 infection. Population-based descriptions of immunocompromised hospitalized patients and their outcomes are limited.

**What is added by this report?**

Immunocompromised patients accounted for 12.2% of all adult COVID-19 hospitalizations among 10 states and had increased odds of ICU admission and in-hospital death compared with nonimmunocompromised patients, irrespective of vaccination status.

**What are the implications for public health practice?**

Known multilayered prevention measures, including nonpharmaceutical interventions, up-to-date COVID-19 vaccination, and therapeutics, can prevent hospitalization and subsequent severe COVID-19 outcomes among immunocompromised persons.

## Footnotes

* Vaccinated patients were defined as those with a positive SARS-CoV-2 test result from a specimen collected ≥14 days after either the second dose of a 2-dose vaccination series or after 1 dose of a single-dose vaccine. When not otherwise specified, vaccinated patients include those who might have received additional or booster doses. Vaccinated patients without additional or booster doses include both those eligible and those not yet eligible for an additional or booster dose. Vaccinated patients with additional booster doses received additional or booster doses on or after August 13, 2021, with a positive SARS-CoV-2 test result from a specimen collected ≥14 days after receipt of additional or booster doses.

†
https://www.cdc.gov/coronavirus/2019-ncov/prevent-getting-sick/prevention.html

§
https://www.cdc.gov/coronavirus/2019-ncov/vaccines/recommendations/immuno.html

¶ https://www.cdc.gov/coronavirus/2019-ncov/need-extra-precautions/people-with-medical-conditions.html; https://www.covid19treatmentguidelines.nih.gov/overview/prioritization-of-therapeutics/

** California, Colorado, Connecticut, Georgia, Michigan, Minnesota, New Mexico, New York, Oregon, and Tennessee. Data from four other states in COVID-NET were not available for this analysis.

†† Excluded admissions for labor/obstetrics, trauma, psychiatric conditions, or inpatient surgery.

§§ Standardized COVID-NET medical chart abstraction identified the following immunocompromising conditions: AIDS or CD4+ count <200, complement deficiency, graft versus host disease, HIV infection, immunoglobulin deficiency/immunodeficiency, immunosuppressive therapy (within 12 months before admission), leukemia, Hodgkin or non-Hodgkin lymphoma, metastatic cancer, multiple myeloma, solid organ malignancy, steroid therapy (within 2 weeks of admission), and transplant history involving hematopoietic stem cells or solid organs.

¶¶
https://www.cdc.gov/media/releases/2021/s0813-additional-mRNA-mrna-dose.html

*** Pre-Delta variant–predominant period = March 1, 2020–June 26, 2021; Delta variant–predominant period = June 27–December 18, 2021; Omicron variant–predominant period = December 19, 2021–February 28, 2022. https://www.cdc.gov/mmwr/volumes/71/wr/mm7116e1.htm

††† 45 C.F.R. part 46.102(l)(2), 21 C.F.R. part 56; 42 U.S.C. Sect. 241(d); 5 U.S.C. Sect. 552a; 44 U.S.C. Sect. 3501 et seq.

§§§ A total of 2,280 patients were excluded: 80 (3.5%) had incomplete chart review, 107 (4.7%) had missing discharge date, 1,441 (63.2%) had a non–COVID-19 related admission (labor/obstetrics [15], inpatient surgery [376], psychiatric condition [509], and trauma [541]), and 652 (28.6%) whose vaccination status did not meet the study's vaccinated or unvaccinated definition.

¶¶¶ ICU admission among unvaccinated patients was adjusted for age, sex, site, variant predominant period, race/ethnicity, hypertension, diabetes, chronic lung disease, cardiovascular disease, renal disease, and obesity. Death among unvaccinated patients was adjusted for age, sex, site, variant predominant period, race/ethnicity, long-term care facility, hypertension, diabetes, chronic lung disease, cardiovascular disease, renal disease, blood disorders, neurologic disease, and rheumatologic/autoimmune condition.

**** ICU admission among vaccinated patients was adjusted for age, sex, site, variant predominant period, race/ethnicity (American Indian or Alaska Native and Asian or Pacific Islander were reclassified to other/unknown because of small numbers), chronic metabolic disease, liver disease, and rheumatologic/autoimmune condition. Death among vaccinated patients was adjusted for age, sex, site, variant predominant period, race/ethnicity, cardiovascular disease, renal disease, and rheumatologic/autoimmune condition.

†††† ICU admission among immunocompromised patients was adjusted for age, sex, site, variant predominant period, rheumatologic/autoimmune condition, and obesity. Death among immunocompromised patients was adjusted for age, sex, site, variant predominant period, hypertension, renal disease, and rheumatologic/autoimmune condition.

§§§§ Death among nonimmunocompromised patients was adjusted for age, sex, site, variant predominant period, hypertension, diabetes mellitus, chronic metabolic disease, cardiovascular disease, renal disease, and neurologic disease.

¶¶¶¶
https://www.cdc.gov/mmwr/volumes/71/wr/mm7112e2.htm

*****
https://www.cdc.gov/coronavirus/2019-ncov/science/science-briefs/indicators-monitoring-community-levels.html

†††††
https://www.cdc.gov/coronavirus/2019-ncov/community/health-equity/race-ethnicity.html
